# Expression of hypothalamic neurohormones and their receptors in the human eye

**DOI:** 10.18632/oncotarget.18358

**Published:** 2017-06-03

**Authors:** Sander R Dubovy, Maria P Fernandez, Jose J Echegaray, Norman L Block, Noriyuki Unoki, Roberto Perez, Irving Vidaurre, Richard K Lee, Mehrdad Nadji, Andrew V Schally

**Affiliations:** ^1^ Florida Lions Ocular Pathology Laboratory, Bascom Palmer Eye Institute, University of Miami, Miller School of Medicine, Miami, Florida, USA; ^2^ Miami Veterans Affairs Medical Center, Miami, Florida, USA; ^3^ Department of Pathology, University of Miami, Miller School of Medicine, Miami, Florida, USA; ^4^ Divisions of Hematology/Oncology, Endocrinology, Department of Medicine, University of Miami, Miller School of Medicine, Miami, Florida, USA; ^5^ Department of Ophthalmology, University of Puerto Rico School of Medicine, San Juan, PR, USA

**Keywords:** eye neuropeptides, ocular endocrinology, hormone-analog therapy

## Abstract

Extrapituitary roles for hypothalamic neurohormones have recently become apparent and clinically relevant, based on the use of synthetic peptide analogs for the treatment of multiple conditions including cancers, pulmonary edema and myocardial infarction. In the eye, it has been suggested that some of these hormones and their receptors may be present in the ciliary body, iris, trabecular meshwork and retina, but their physiological role has yet to be elucidated. Our study intends to comprehensively demonstrate the expression of some hypothalamic neuroendocrine hormones and their receptors within different retinal and extraretinal structures of the human eye. Immunofluorescence, Western blot analysis, and RT-PCR were used to evaluate the qualitative and quantitative expression of Luteinizing Hormone Releasing Hormone (LHRH), Growth Hormone Releasing Hormone (GHRH), Thyrotropin Releasing Hormone (TRH), Gastrin Releasing Peptide (GRP) and Somatostatin as well as their respective receptors (LHRH-R, GHRH-R, TRH-R, GRP-R, SST-R1) in cadaveric human eye tissue and in paraffinized human eye tissue sections. The hypothalamic hormones LHRH, GHRH, TRH, GRP and Somatostatin and their respective receptors (LHRH-R, GHRH-R, TRH-R, GRPR/BB2 and SST-R1), were expressed in the conjunctiva, cornea, trabecular meshwork, ciliary body, lens, retina, and optic nerve.

## INTRODUCTION

It has been known since the 1930's that the anterior pituitary gland secretes several hormones which not only stimulate the thyroid, the gonads, and the adrenal cortex, but also regulate various physiologic processes. However, the mechanisms of the control of pituitary function itself were not understood. In the 1950’s, G.W. Harris assembled anatomical and physiological evidence suggesting that the hypothalamic portion of the brain secreted neurohormones that traversed the hypophyseal portal vessels to regulate secretion of pituitary hormones [1]. The work to prove Harris's neurohormonal theory extended over 30 years, and one of us (A.V.S.) played a major part in this endeavor. The discovery, isolation, structural identification, chemical synthesis, and information gathered from animal and human studies, regarding several of these hypothalamic hormones, established their regulatory function in the process of mediation of release of various anterior pituitary hormones [2–12].

Hypothalamic hormones are now known to influence growth, reproduction, lactation, metabolism, gastrointestinal function, and the response to stress, by initiating and regulating the hypothalamic-pituitary axis (HPA). This mediation involves a set of endocrine feedback mechanisms that greatly influence reproductive processes and the aforementioned metabolic activities. These regulatory effects are exerted by endocrine hormone ligands binding directly to specific receptors in appropriate target organs. Importantly, the expression of these receptors also extends to organs outside the HPA. Thus, interactions between agonistic and antagonistic hypothalamic hormone ligands and extrapituitary receptors may influence the physiology of host organs or the pathologic progression of a tumor or other lesion [13–16]. The development of synthetic analogs of hypothalamic hormones such as Luteinizing Hormone Releasing Hormone (LHRH; also called Gonadotropin Releasing Hormone, or GnRH) [17], Growth Hormone Releasing Hormone (GHRH) [18] and others has led to innovative experimental findings and to various clinical applications such as the treatment of benign prostatic hyperplasia [19] and prostate cancer [20, 21]. In addition, multiple preclinical and experimental studies have suggested that targeted cytotoxic and radiolabeled peptide conjugates, specific to hypothalamic hormone receptors, can be powerful tools for diagnosis, for primary therapy or, as adjuvants, to treat neoplastic lesions in extrapituitary organs such as breast [22–25], ovaries, endometrium [22], prostate, colon [26], liver [27] urinary bladder [28] and lung [29]. Moreover, GHRH-agonist-based therapy has recently been shown to serve cardioprotective and regenerative roles in patients with myocardial infarction [30–32].

GRP belongs to the bombesin-like peptide family, and is not a classical hypothalamic-hypophyseal regulatory hormone since it plays only a perfunctory role in the mediation of pituitary hormone release. However, GRP/bombesin-like immunoreactivity is widely distributed in mammalian brain, especially the hypothalamus, GI tract and in human fetal lung [33].

The expression of hypothalamic hormones and their receptors in the mammalian eye has been previously reported, mainly using rat models and mostly limited to retinal tissue [34–39]. Somatostatin receptors have been previously described in the human retina and other ocular structures [40–43] as has TRH, which has also been identified in human retina [44]. However, the presence of other neuropeptides and their receptors has yet to be systematically or extensively described in the multiple sub-portions of ocular anatomy.

With the onset of the transformational medical role that anti-VEGF therapy has exerted in the field of diabetic retinopathy, somatostatin analogs have been presented as a potential therapeutic alternative for these patients [45] as well as for patients with age-related macular degeneration [46]. Further, antagonistic effects of GHRH and cAMP response element-binding-protein analogs have been suggested to mediate reduction of ocular inflammation in animal models [47]. TRH has been indirectly associated with intraocular pressure elevation [48]. To our knowledge, mechanisms for the role of these neuropeptides in ocular physiology and pathology in the human eye remain to be defined. These investigations, of course, would be abetted and would need to be preceded by such a systematic verification of the presence of these neuropeptides and their receptors.

In this study, we used immunofluorescence staining, Western blot analysis, and RT-PCR to comprehensively identify the expression of the following hypothalamic neuropeptide ligands: LHRH, GHRH, TRH, somatostatin, and GRP as well as the presence of their respective receptors (LHRH-R, GHRH-R, TRH-R, SST-R1 and GRP-R) in the various human eye structures. The structures we included are the conjunctival epithelium, corneal epithelium, corneal endothelium, trabecular meshwork, ciliary body muscle, ciliary body non-pigmented epithelium, lens epithelium, retinal nerve fiber layer, neural retina and optic nerve. The presence of these hormones and their receptors in these structures suggests their potential role in human ocular physiology and in the pathogenesis of ocular disease and may also provide molecular-based targets for the treatment of neoplastic and non-neoplastic ophthalmic diseases.

## RESULTS

### Luteinizing hormone releasing hormone and its receptor

**Figure 1:**
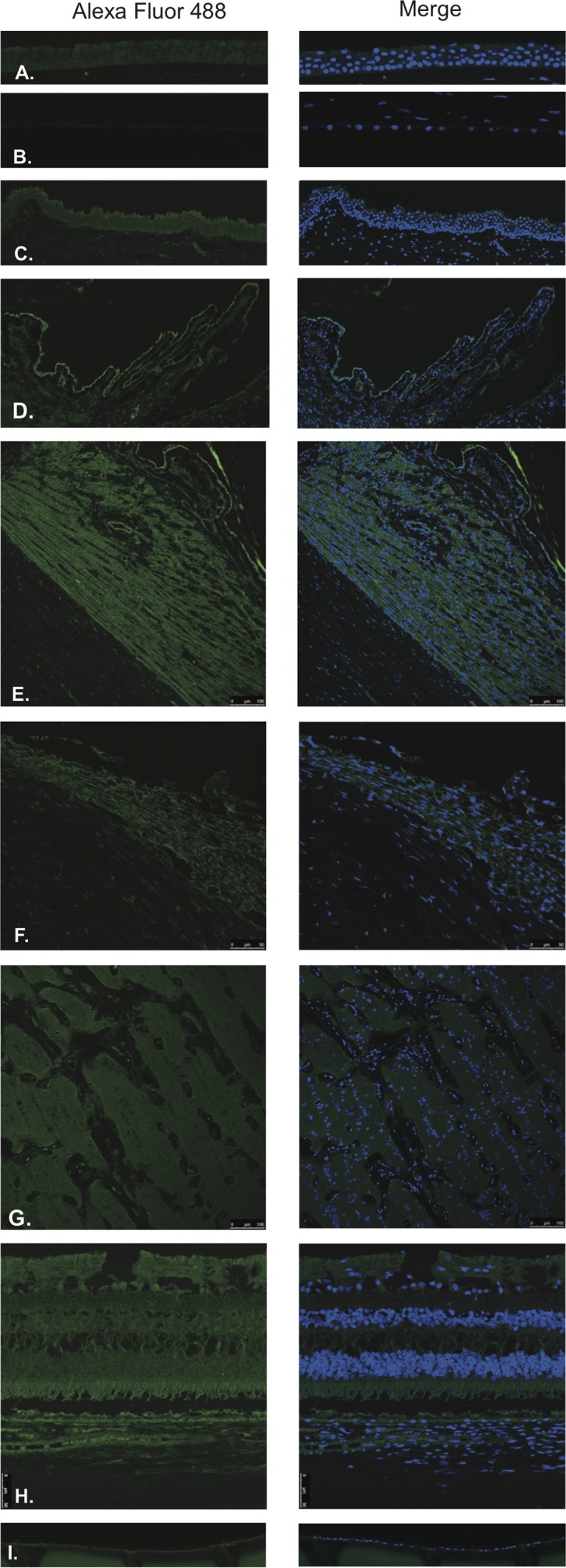
Luteinizing hormone releasing hormone (LHRH)

**Figure 2:**
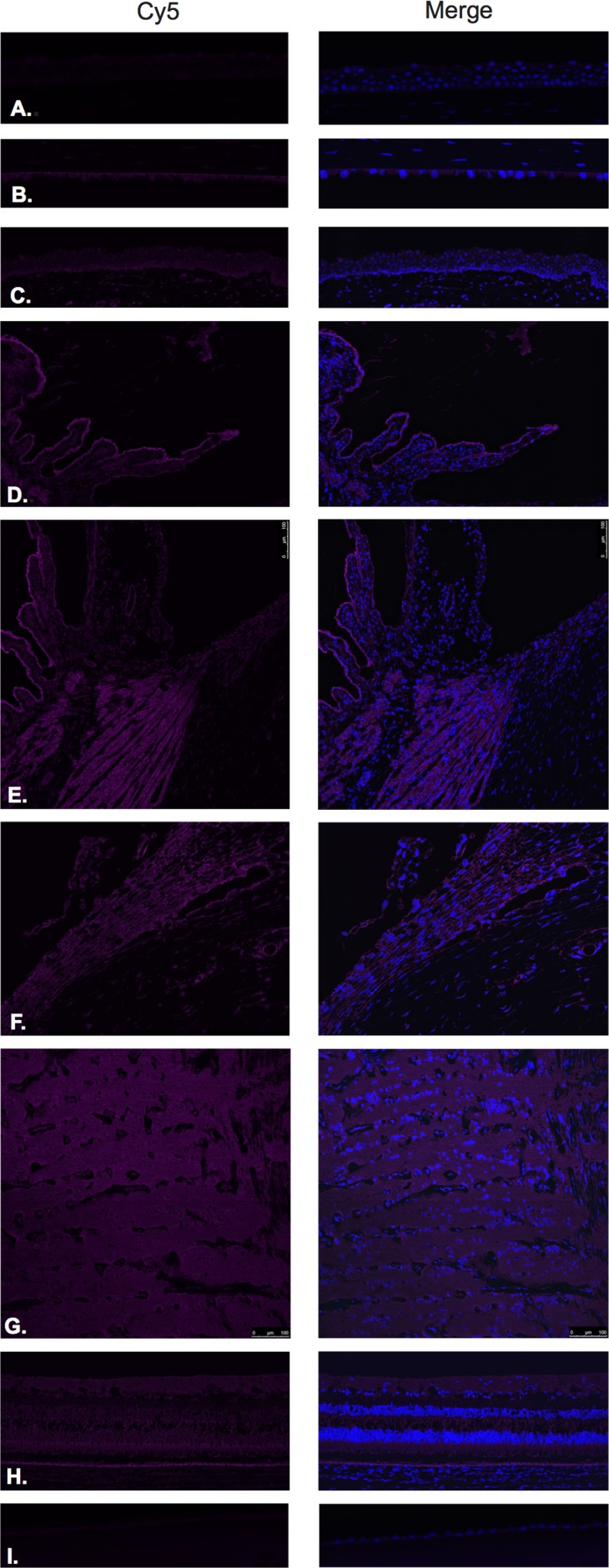
Luteinizing hormone releasing hormone receptor (LHRH-R)

LHRH (Figure 1) and LHRH-R (Figure 2) were identified by Immunofluorescence in paraffin-embedded human eyes using anti-LHRH and anti-LHRH-R antibodies.

LHRH was found to be expressed in the corneal epithelium (Figure 1A), conjunctival epithelium (Figure 1C), ciliary body non-pigmented epithelium (Figure 1D), ciliary body muscle (Figure 1E) trabecular meshwork (Figure 1F), optic nerve (Figure 1G), neural retina (Figure 1H) and lens epithelium (Figure 1I). No expression of LHRH was observed in the corneal endothelium (Figure 1B). Expression in the corneal epithelium, conjunctival epithelium and lens epithelium was weaker than the expression in the other aforementioned structures. LHRH was found to be diffusely expressed throughout all layers of the neural retina and RPE.

LHRH-R was found to be expressed in the ciliary body non-pigmented epithelium (Figure 2D), ciliary body muscle (Figure 2E), trabecular meshwork (Figure 2F), optic nerve (Figure 2G) and neural retina (Figure 2H). Very weak signal was found to be present in the corneal endothelium (Figure 2B) conjunctival epithelium (Figure 2C) and neural retina (Nerve fiber layer (NFL), inner nuclear layer, outer plexiform layer, outer nuclear layer and photoreceptors). Stronger signal was identified at the level of the RPE (Figure 2H). No expression of LHRH-R was observed in the corneal epithelium (Figure 2A) or lens epithelium (Figure 2I).

LHRH-R expression was also detected by Western blot in unfixed cadaveric human eye tissues (Figure 11). Cadaveric conjunctival, corneal epithelium, retinal, and optic nerve tissue samples were dissected and equal amounts of protein were loaded for each sample. We detected major bands at 42 kD corresponding to LHRH receptor, for all conjunctiva, corneal epithelium, neural retina, and optic nerve tissues. Actin was used for internal control for all samples tested.

### Growth hormone releasing hormone and its receptors

**Figure 3:**
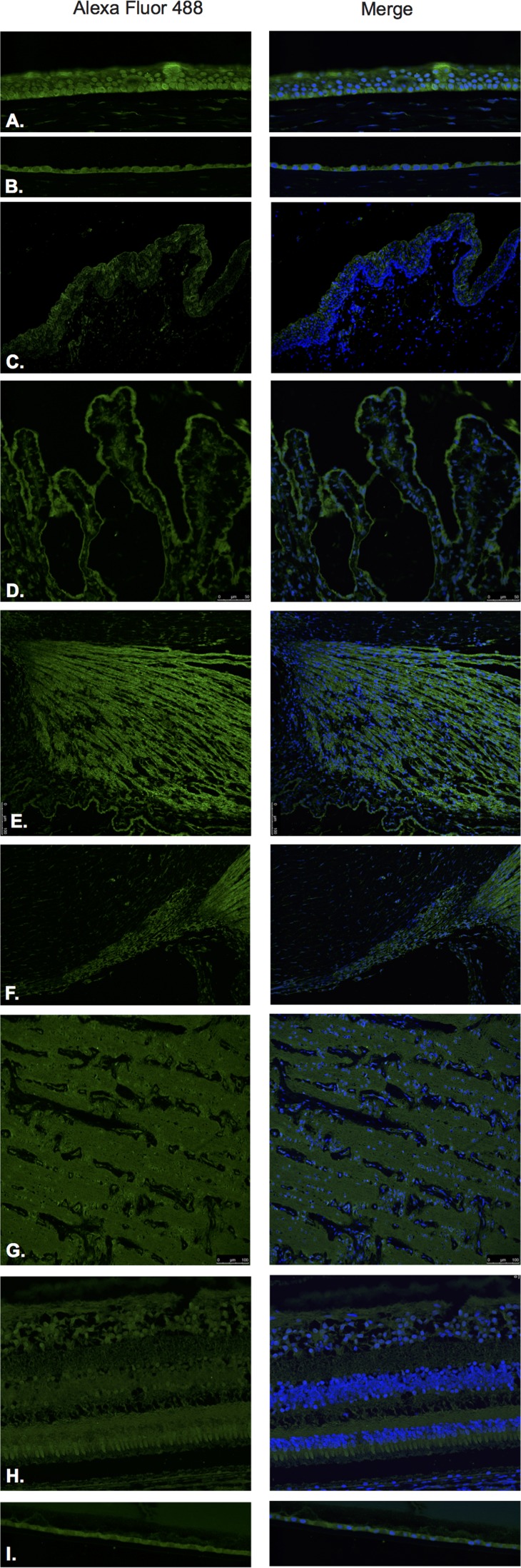
Growth hormone releasing hormone (GHRH)

**Figure 4:**
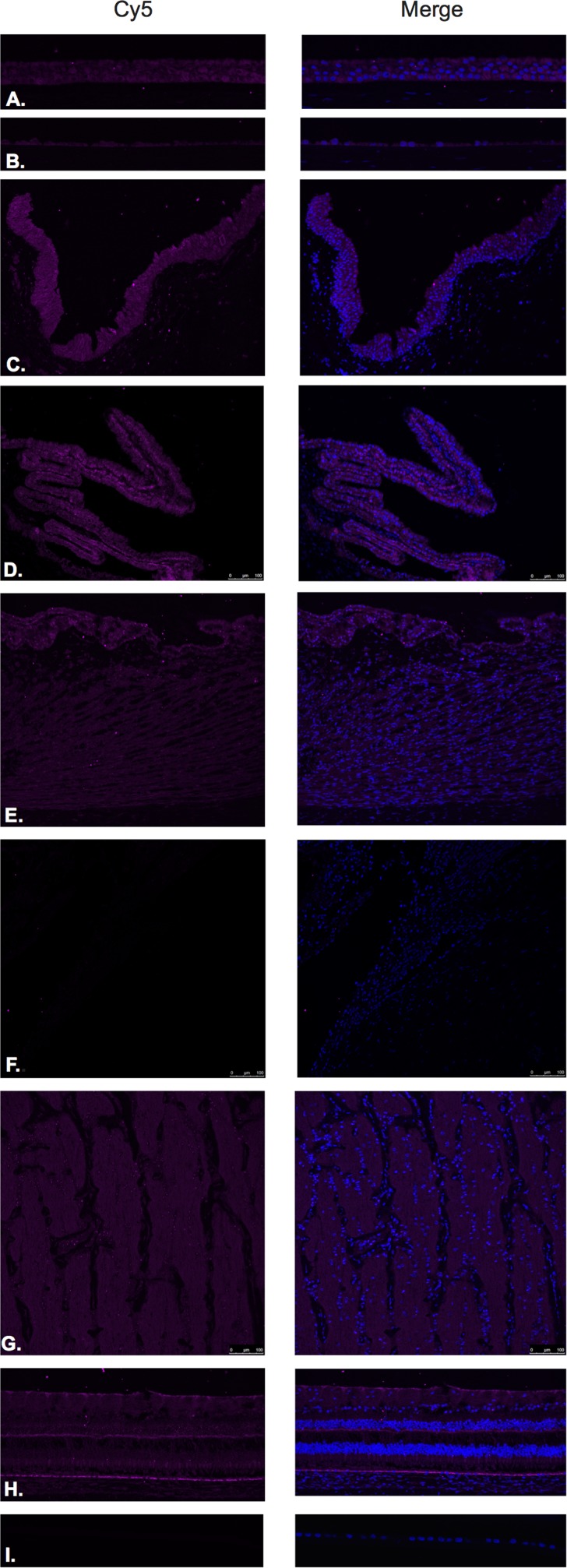
Growth hormone releasing hormone receptor (GHRH- R)

GHRH (Figure 3) and GHRH-R (Figure 4) were identified by Immunofluorescence in paraffin-embedded human eyes using anti-GHRH and anti-GHRH-R antibodies.

GHRH was found to be expressed in the corneal epithelium (Figure 3A), corneal endothelium (Figure 3B) conjunctival epithelium (Figure 3C), ciliary body non-pigmented epithelium (Figure 3D), ciliary body muscle (Figure 3E) trabecular meshwork (Figure 3F), optic nerve (Figure 3G), neural retina (Figure 3H) and lens epithelium (Figure 3I). GHRH was found to be diffusely expressed throughout all layers of the neural retina with no expression being observed at the level of the RPE (Figure 3H).

GHRH-R was found to be expressed in the corneal epithelium (Figure 4A), corneal endothelium (Figure 4B), conjunctival epithelium (Figure 4C), ciliary body non-pigmented epithelium (Figure 4D), ciliary body muscle (Figure 4E), optic nerve (Figure 4G) and neural retina (Figure 4H). Weak signal was found to be present in the corneal endothelium (Figure 4B) ciliary body muscle (Figure 4F), optic nerve (Figure 4G), outer plexiform layer, outer nuclear layer and photoreceptors in the neural retina. Stronger signal was identified at the level of the NFL, outer aspect of the inner nuclear layer and RPE. (Figure 4H). No expression of GHRH-R was observed in the trabecular meshwork (Figure 4E) or lens epithelium. (Figure 4I).

Western blot analysis using anti-GHRH-R antibody demonstrated major bands at molecular weight (39.1 kD) corresponding to GHRH-R splice variant-1 (SV-1) in corneal epithelium, neural retina, and optic nerve (Figure 11). There was a 50 kD band present in all tissues (conjunctiva, corneal epithelium, neural retina and optic nerve) corresponding to unglycosylated GHRH-R. Additionally, we detected expression for GHRH-R proteins at higher molecular weights (63.7 kD) corresponding to Pituitary Type GHRH receptor (pGHRH-R) in all four tested samples: conjunctiva, corneal epithelium, neural retina, and optic nerve. These major bands correspond to the glycosylated form of the receptor. Actin was used for internal control for all samples tested.

### Thyrotropin releasing hormone and its receptor

**Figure 5:**
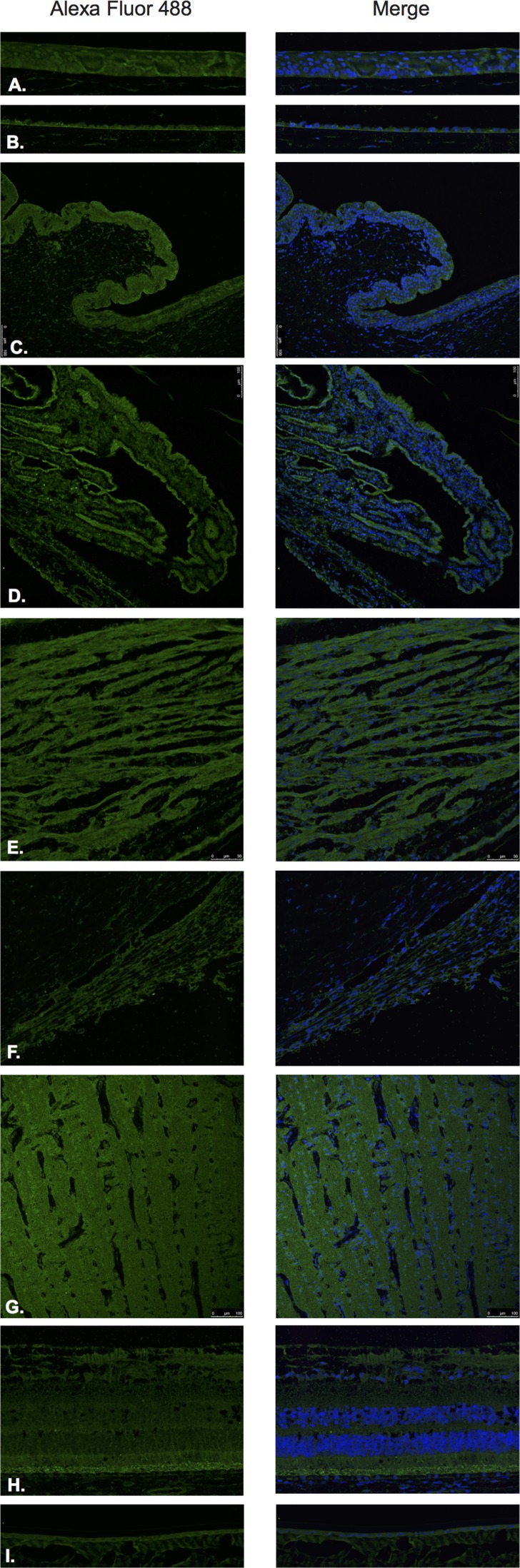
Thyrotropin releasing hormone (TRH)

**Figure 6:**
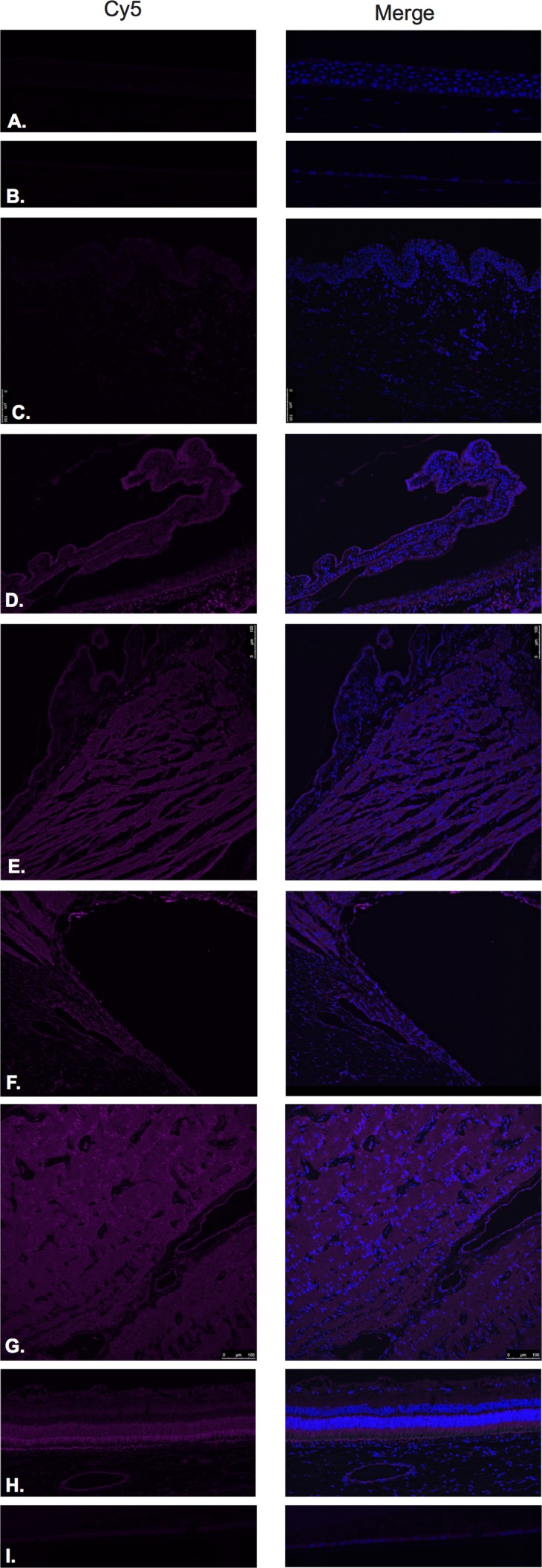
Thyrotropin releasing hormone receptor (TRH-R)

TRH (Figure 5) and TRH-R (Figure 6) were identified by Immunofluorescence in paraffin-embedded human eyes using anti-TRH and anti-TRH-R antibodies.

TRH was detected in the corneal epithelium (Figure 5A), corneal endothelium (Figure 5B), conjunctival epithelium (Figure 5C), ciliary body non-pigmented epithelium (Figure 5D), ciliary body muscle (Figure 5E), trabecular meshwork (Figure 5F), optic nerve (Figure 5G), neural retina (Figure 5H) and lens epithelium (Figure 5I). In the neural retina, TRH was found to be expressed in the NFL, inner and outer nuclear layers, inner and outer plexiform layers with stronger immunoreaction in the inner and outer segments of photoreceptors (Figure 5H).

TRH-R was found to be expressed in the ciliary body non-pigmented epithelium (Figure 6D), ciliary body muscle (Figure 6E), optic nerve (Figure 6G) and neural retina (Figure 6H). No expression was observed in the corneal epithelium (Figure 6A), corneal endothelium (Figure 6B), conjunctival epithelium (Figure 6C) or lens epithelium (Figure 6I). Weak signal was detected in the trabecular meshwork (Figure 6F). In the neural retina, TRH-R was strongly expressed in the outer nuclear layer and inner segments of photoreceptors (Figure 6H).

Western blot was performed on these tissues but no clear signals were obtained for TRH-R (Results not shown).

### Somatostatin and its receptor (SST-R1)

**Figure 7:**
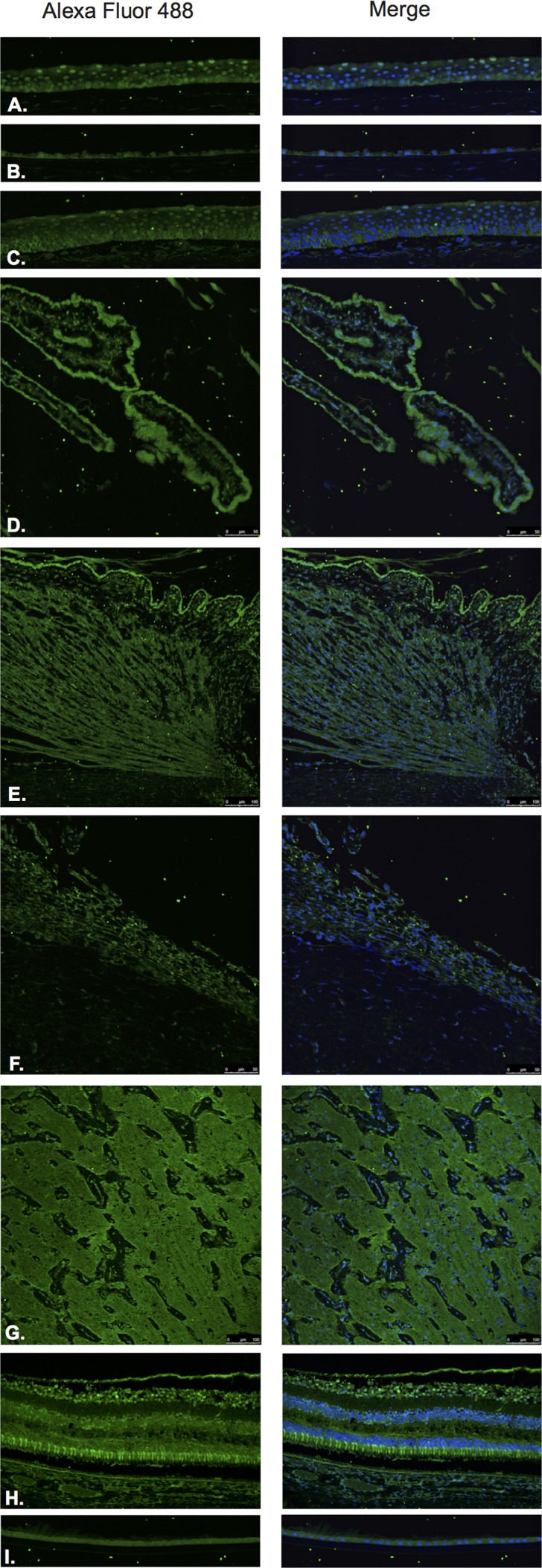
Somatostatin

**Figure 8:**
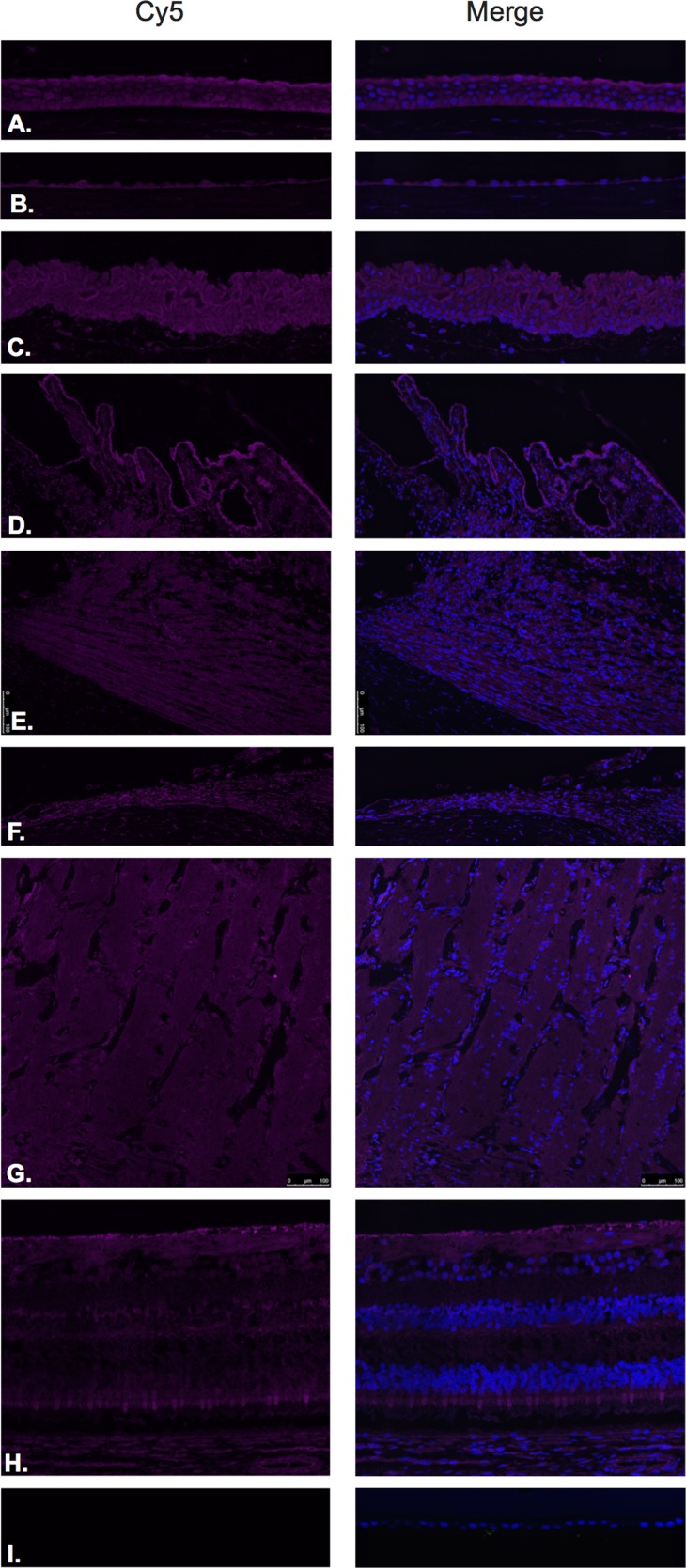
Somatostatin receptor 1(SST-R1)

Somatostatin hormone (Figure 7) and its predominant receptor in the CNS, SST-R1 (Figure 8) were identified by Immunofluorescence in paraffin-embedded human eyes using anti-Somatostatin and anti-Somatostatin Receptor 1 antibodies.

Somatostatin was detected in corneal epithelium (Figure 7A), corneal endothelium (Figure 7B), conjunctival epithelium (Figure 7C), ciliary body non-pigmented epithelium (Figure 7D), ciliary body muscle (Figure 7E), trabecular meshwork (Figure 7F), optic nerve (Figure 7G), neural retina (Figure 7H) and lens epithelium (Figure 7I). In the retina, somatostatin was found to be expressed in the NFL, ganglion cell layer, inner and outer nuclear layers, inner and outer plexiform layers and RPE (Figure 7H).

SST-R1 was expressed within the corneal epithelium (Figure 8A), corneal endothelium (Figure 8B), conjunctival epithelium (Figure 8C), ciliary body non-pigmented epithelium (Figure 8D), ciliary body muscle (Figure 8E), trabecular meshwork (Figure 8F), optic nerve (Figure 8G) and neural retina (Figure 8H). No expression was observed in the lens epithelium (Figure 8I). In the neural retina, SST-R1 was strongly expressed in the NFL, inner nuclear layer and inner segments of photoreceptors (Figure 8H).

SST-R1 expression was also detected by Western blot in cadaveric human eye tissues (Figure 11). Cadaveric conjunctival, corneal epithelium, retinal, and optic nerve tissue samples were dissected and equal amounts of protein were loaded for each sample. Major bands were demonstrated at 48 kD corresponding to SST-R1 for all conjunctiva, corneal epithelium, retina, and optic nerve tissues. Actin was used for internal control for all samples tested.

### Gastrin releasing peptide and its receptor

**Figure 9:**
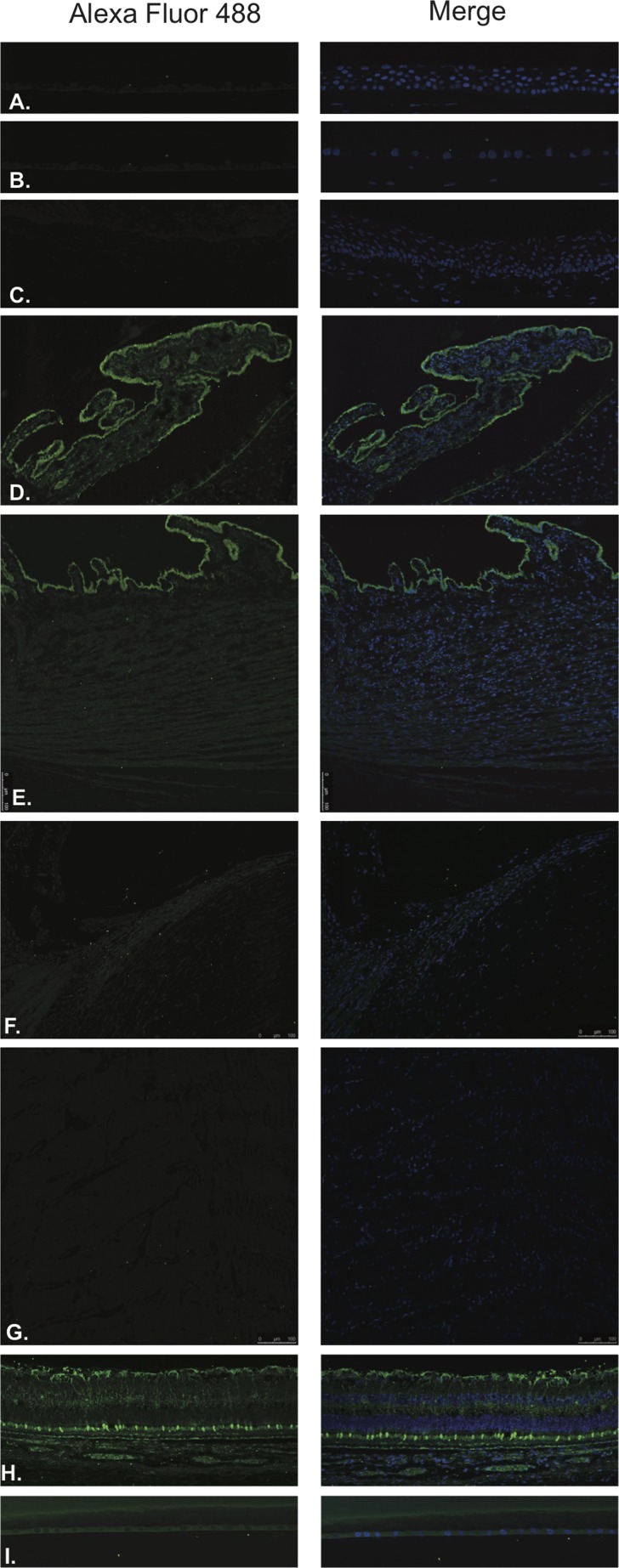
Gastrin releasing peptide (GRP)

**Figure 10:**
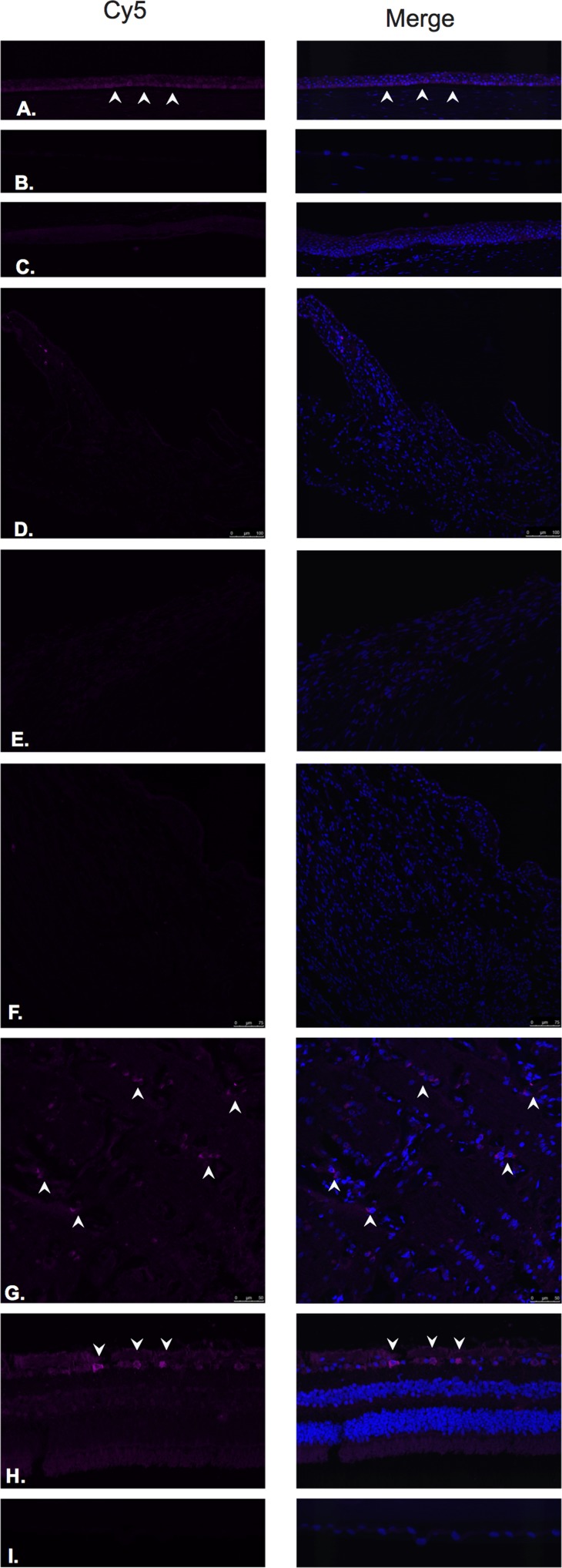
Gastrin releasing peptide receptor (GRP-R)

GRP (Figure 9) and GRP-R/BB2, receptor for GRP/mammalian bombesin, (Figure 10) were identified by Immunofluorescence in paraffin-embedded human eyes using anti-GRP and anti-GRP Receptor antibodies.

**Figure 11:**
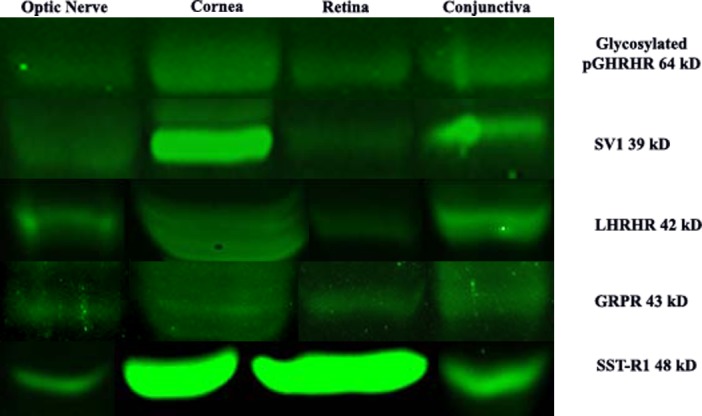
Western blot analysis of neuroendocrine receptor expression in optice nerve, cornea, retina and conjunctiva

GRP was detected in ciliary body non-pigmented epithelium (Figure 9D), retina (Figure 9H) and lens epithelium (Figure 9I). Weak signal was detected in the ciliary body muscle (Figure 9E) and trabecular meshwork (Figure 9F). In the neural retina, GRP was found to be strongly and diffusely expressed in the NFL, ganglion cell layer, inner and outer nuclear layers, inner and outer plexiform layers, inner photoreceptor segments and at the level of the RPE (Figure 9H). No expression was observed in the corneal epithelium (Figure 9A), corneal endothelium (Figure 9B), conjunctival epithelium (Figure 9C) or optic nerve (Figure 9G).

Expression of GRP-R was detected in the corneal epithelium (Figure 10A) mostly at the level of basal epithelial layer. GRP-R was also identified in a few cells within the optic nerve substance (Figure 10G) and at the level of the ganglion cell layer in the retina (Figure 10H) No expression was observed in the corneal endothelium (Figure 10B), conjunctival epithelium (Figure 10C), ciliary body non-pigmented epithelium (Figure 10D), ciliary body muscle (Figure 10E), trabecular meshwork (Figure 10F) or lens epithelium (Figure 10I).

The presence of GRP-R protein was further detected in ocular tissue using Western blot analysis (Figure 6). All tissues that were sampled (conjunctiva, corneal epithelium, retina, and optic nerve) showed GRP-R expression with major bands detected at 43 kD corresponding to the GRP receptor. Actin was used for internal control for all samples tested.

### Expression of hypothalamic neurohormones and receptor mRNAs in human retina tissue

Pairs of retinal tissue (right and left) were collected from five pairs of unfixed cadaveric eyes. Samples (*N* = 10) were individually homogenized and mRNA isolated from each. We analyzed each sample, in triplicate, using one-step RT-qPCR with SYBR green chemistry. The expression of each target gene was normalized to the geometric mean of the expression of actβ, β-2M, and GAPDH from the corresponding sample (δδCt method). All retina samples were found to express growth hormone-releasing hormone (GHRH), its receptor (GHRH-R), and a functional splice variant of GHRH-R (SV-1) (Supplementary Table 1). All samples were also found to express gastrin-releasing peptide (GRP) and its receptor (GRP-R); luteinizing hormone-releasing hormone (LHRH) and its receptor (LHRH-R); somatostatin (SST) and SST receptors 1 and 2 (SST-R1 and SST-R2); and thyrotropin-releasing hormone (TRH) and its receptor (TRH-R) (Supplementary Table 1). Sequences for the target mRNA were obtained using the provided accession number in the NCBI database (Supplementary Table 2).

## DISCUSSION

The present study systematically demonstrates the expression of LHRH, GHRH, TRH, somatostatin and GRP and their receptors in retinal and extraretinal structures of the human eye, using different techniques, including RT-PCR, Western blot and Immunofluorescence. In addition, we have shown that the expression of these neuroendocrine hormones and their receptors varies throughout different anatomic locations within the eye.

This data may have significant clinical implications by contributing to the better understanding of neuroendocrine influences within the eye. Establishing the presence of hypothalamic hormones and their receptors for each ocular structure also provides a distribution map for these peptides and thereby provides a key for the better understanding of physiologic, pathophysiologic and clinical implications such as the ones that have already been described in the literature [39, 40].

Several studies have demonstrated the wide clinical and therapeutic role that these neuropeptides and their receptors may play in various human diseases including cancer, myocardial infarction and pulmonary edema. Analogs of these peptides and their receptors have been used to manipulate the growth of certain tumors such as breast and prostate cancers. New treatment alternatives for patients with cancer may be based on the ability of these analogs to alter the course and progression of the involved tumors.

Previous studies have described the presence and possible effects of TRH and somatostatin in the eye, specifically in the retina, using mouse and other mammalian models including rabbits, cats, and primates. Few studies have demonstrated the presence of these neuropeptides in the human retina using techniques other than immunofluorescence. To our knowledge, this is the first time that immunofluorescence studies have been used to demonstrate the presence of LHRH, GHRH, TRH, somatostatin, GRP and their receptors, using formalin fixed paraffin embedded tissue, in the human eye. Minor differences were found between the results obtained from RT-PCR, Western blot and Immunofluorescence for the expression of LHRH, GHRH, Somatostatin, GRP and their receptors. No clear band or positive signal was identified using the Western blot technique for TRH receptor. However, RT-PCR and immunofluorescence demonstrated its presence in the neural retina and optic nerve.

These techniques may help to evaluate and characterize the presence of these peptides and their role in other ophthalmic diseases such as Ocular Surface Squamous Neoplasia (OSSN), intraocular tumors and diabetic retinopathy, among others. These neuropeptides may play a significant role in multiple ocular physiologic mechanisms, including regulatory roles in ocular surface homeostasis, secretion or absorption of aqueous humor, and function of retinal fiber layer, ganglion cell layer, photoreceptor and optic nerve, amongst others. The use of synthetic neuropeptide analogs that have been developed to treat a number of diseases [40-41) may now be extended, with appropriate preliminary clinical evaluation, to patients with ophthalmic diseases, given that we have shown that hypothalamic target receptors exist throughout the eye. Previous studies have demonstrated that the localization, distribution and expression pattern of these receptors and their subtypes may differ depending on the pathology present and the targeted tissue [46, 47], however, these have generally been disease specific. Our findings support the merit of further verification, elucidation, and mapping of the distribution of these neuropeptides and their respective receptors to further delineate the role that analogs of these hormonal peptides may play in models of various eye diseases.

## MATERIALS AND METHODS

This study was approved by the Institutional Review Board (IRB) at the University of Miami, Miller School of Medicine.

### Immunofluorescence staining protocol

Two formalin-fixed paraffin embedded eyes, from patients without any clinical history of intraocular disease and having no histological evidence of intraocular pathology, were selected from the Florida Lions Ocular Pathology Laboratory archives. Four-micron sections were cut from the paraffin embedded blocks. Slides were de-paraffinized and rehydrated with xylene and graded series of ethanol. Antigen retrieval was performed with Trilogy antigen retrieval solution (Cell Marque, Rocklin, California) and subsequently washed in 0.5% Triton and PBS. Blocking was performed with Background Terminator (Biocare Medical, Concord, California). The following primary polyclonal antibodies (Abcam) were used: Anti-LHRH antibody (ab5617) Anti-LHRH Receptor antibody (ab183079), Anti-GHRH antibody (ab8899), Anti-GHRH Receptor antibody (ab76263), Anti-TRH antibody (ab174714), Anti-TRH Receptor antibody (ab151510), Anti-somatostatin antibody (ab183855), Anti-somatostatin Receptor-1 antibody (ab2366), Anti-GRP antibody (ab202123) and Anti-GRP Receptor antibody (ab188821). Primary antibody was diluted in 0.5% Triton and PBS and left overnight at 4°C. After washing, the sections were incubated with the secondary antibody for 120 minutes. Alexa Fluor 488 (ab150077) was used with Anti-LHRH antibody, Anti-GHRH antibody, Anti-TRH antibody, Anti-GRP antibody and Anti-somatostatin antibody. Cy5 (ab97077) was used with Anti-LHRH Receptor antibody, Anti-GHRH Receptor antibody, Anti-TRH Receptor antibody, Anti-GRP Receptor antibody and Anti-somatostatin Receptor-1 antibody. Slides were then rinsed in PBS, mounted in Vectashield (Vector Laboratories) and sealed with clear nail varnish. Stained slides were stored at 4°C. Sections were visualized with a Leica confocal scanning microscope. Negative control assays were performed by omission of the primary antibodies. Null staining of these negative control sections confirmed the specificity of the staining (Data not shown).

### Western blot analysis

Cadaveric human eyes were obtained from the Florida Lions Eye Bank within 48 hours of death. Only deceased subjects without documented history of ophthalmic diseases or ophthalmic surgery were considered. Eyes were dissected and conjunctival tissue, corneal epithelium, retinal tissue, and optic nerve substance were isolated and snap frozen in dry ice.

For Western blot (WB) analysis, equal amounts of protein were loaded for all samples. Actin was run as an internal control for all samples. The following antibodies were utilized for WB assays: LHRH-R (Abcam ab-58561), GHRH-R/SV1 (Abcam ab28692), GRP-R (Santa Cruz sc-26836), SST-R1 (Santa Cruz sc-25675), and Actin (Sigma A5441).

### SYBR green-based RT-PCR primer design

Gene expression was determined using qRT-PCR. All RNA targets were analyzed using custom designed oligonucleotide primers designed for use in SYBR green (ThermoFisher Scientific, Waltham, MA)based qRT-PCR. The assays were designed using extremely strict parameters in order to exclude non-human templates and target regions of low energy secondary structures, thus maximizing both specificity and sensitivity. All assays were determined to produce a single product, which was verified as the human target of interest by DNA sequencing.

Transcript specific primers were designed using the Beacon Designer software suite (PREMIER Biosoft, Palo Alto, California) with modified parameters. Primer searches were limited to regions on mRNA sequences (Refseqs), which were not homologous to the equivalent mRNA from mice (*Mus musculus*). The resulting human-specific sequences were screened for regions of stable secondary structures (∆G < −3.0 Kcal/mol), which were excluded from our primer search. Primer searches were optimized for reverse transcription at 52°C and fast cycling PCR with single step annealing/extension at 57°C. Primer hairpin energy was limited to ∆G = −3.0 Kcal/mol; dimer energies were limited to ∆G = −4.0 Kcal/mol. Dimers including the last 3 bases of the 3’ end of the primer were limited to ∆G = −2.0 Kcal/mol. Primers were designed to result in amplicons of 75-200 bp in length. Primer pairs that were less than 98% efficient were excluded. All primers used produce a single product of predictable and reproducible melting temperature (Tm). All primers were optimized and verified by sequencing the corresponding amplicons.

### Real-time quantitative reverse-transcription polymerase chain reaction (qRT PCR)

Gene expression analysis was conducted using one-step qRT-PCR with SYBR green chemistry. This method conducts the reverse transcription reaction and PCR in a single tube format from 20ng total RNA template. The production of the PCR amplified gene product is monitored using the fluorescence resulting from the binding of SYBR green to the double stranded DNA amplicons. Reactions were conducted in a CFX96 Real-Time System using the One-Step SYBR Green qRT-PCR reaction kit (Bio-Rad, Hercules, California). Reactions were conducted in triplicate and normalized to three internal standard genes using the δδCt method.

## SUPPLEMENTARY MATERIALS FIGURES AND TABLES



## Abbreviations

LHRH: Luteinizing Hormone Releasing Hormone
LHRH-R: Luteinizing Hormone Releasing Hormone Receptor
GHRH: Growth Hormone Releasing Hormone
GHRH-R: Growth Hormone Releasing Hormone Receptor
TRH: Thyrotropin Releasing Hormone
TRH-R: Thyrotropin Releasing Hormone Receptor
GRP: Gastrin Releasing Peptide
GRP-R: Gastrin Releasing Peptide Receptor
SST: Somatostatin
SST-R1: Somatostatin Receptor-1
