# Supplementary material for: Expression of hypothalamic neurohormones and their receptors in the human eye
Source: Oncotarget. 2017 Jun 3;8(40):66796–814. doi: 10.18632/oncotarget.18358 (PMC5620137; doi:10.18632/oncotarget.18358)
Supplement: Supplementary file 1 [file oncotarget-08-66796-s001.pdf]

Expression of hypothalamic neurohormones and their receptors in the human eye

SUPPLEMENTARY MATERIALS

Supplementary Table 1: Representation of the mean expression and mean amplification cycle (Cq) for each target gene analyzed.

Target	Mean Expression (N=10)	SEM	Mean Cq (N=30)	Cq SEM
GHRH	0.77681	0.11635	30.2	0.114
GHRH-R	1.50136	0.20273	28.4	0.083
GRP	0.70488	0.09335	24.5	0.068
GRP-R	0.59107	0.07203	27.1	0.087
LHRH	0.88985	0.11420	30.6	0.092
LHRH-R	1.13200	0.12814	35.7	0.091
SST	0.85006	0.10175	27.0	0.062
SSTR1	1.37465	0.15949	25.4	0.074
SSTR2	1.56596	0.17039	25.5	0.068
SV1	1.45217	0.16512	24.2	0.054
TRH	0.41915	0.05861	21.7	0.083
TRH-R	0.93239	0.09960	26.2	0.057

Supplementary Table 2: Details of the oligonucleotide primers developed for the analysis of the target genes in the retina tissue samples. Sequences for the target mRNA can be found using the provided accession number in the NCBI database. All primers are listed 5' to 3'.

Target Gene	Accession Number	Product Length	Product Tm	5'- Sense Primer – 3'<br>5'- Anti-sense Primer – 3'	Position
Control (Housekeeping)					
ActB	NM_001101	89	72.1	CCCACTTCTTAAGGA	1,516
				CATTACATAATTTACACGAAAGC	1,604
B2M	NM_004048	75	66.8	CAACATCAACATCTTGGTC	686
				GCACGCTTAACTATCTTAAC	760
GAPDH	NM_002046	114	74.5	TGAGAAGTATGACAACAGC	513
				ATGAGTCCTTCCACGATA	626
Target Neuropeptides and Receptors					
GHRH	NM_021081	108	75.4	GATGAAGATTCCTCCTGT	323
				AATTTTATTGTATTTTCAAAGG	430
GHRH-R	AY557192	75	73.4	TCAGTATTGGCGTCTCTC	966
				AAGTTGAAGATGATGTAGT	1040
SV1 (GHRH-R)	AF282259	516	87	GAGAGGGAAGGAGTTGTG	291
				AGAACCAGCCACCAGAAG	806
GRP	NM_001012512	90	72	CTTGACTAAATTCGTGATT	652
				GCATTAATTGGAAGACTC	741
GRP-R	NM_005314	79	72.4	CTACCCACTTTAAACCTC	239
				TTTACTAAGAACTTTTGGC	317
LHRH	NM_000825	77	70.5	CCTTTGTGGAAGTTATGTATG	410
				CAGACCTATCAAGAGTTCAA	486
LHRH-R	NM_000406	75	70.7	GAATAACTATCCAGCACTCA	811
				TTCAAATTGGGACCACTTA	885
SST	NM_001048	108	78	CTGGAAGACTTTCACATC	449
				AAGACTTGGAGGATTAGG	556
SSTR-1	NM_001049	120	74.7	TTAGTATGCTGGCTTGTA	3,822
				AATGAGTGAACCTGAGAA	3,941
SSTR-2	NM_001050	77	71.6	CCACTTCTCAGATATAGTC	2,261
				ATACAATGATGGATGACC	2,337
TRH	NM_007117	78	76.2	TTGTGGTCTAAGGATGTC	1,193
				TGGTCAGGGATCTAACTA	1,270
TRH-R	NM_003301	77	74.8	GGCAGTGGTTGTAATTCT	893
				TGGAGAGAAATGAGTTGAC	969
